# Effect of Thermal Aging on Mechanical Properties and Morphology of GF/PBT Composites

**DOI:** 10.3390/polym15183798

**Published:** 2023-09-18

**Authors:** Xiuqi Xu, Jiangang Deng, Siyu Nie, Zhenbo Lan, Zhuolin Xu

**Affiliations:** 1 Department of Electrical Engineering and Automation, China Three Gorges University, Yichang 443002, China; xiuqi_xu2001@163.com; 2 Wuhan Nari Limited Liability Company of State Grid Electric Power Research Institute, Wuhan 430074, China; lan15926378682@163.com (Z.L.); njzlx22@163.com (Z.X.); 3 State Grid Electric Power Research Institute, Nanjing 211103, China; 4 School of Electrical Engineering and Telecommunications, University of New South Wales, Sydney, NSW 2052, Australia; runinininini@gmail.com

**Keywords:** PBT, glass fiber, thermal aging, mechanical properties, failure mechanism

## Abstract

The effects of thermal aging at 85~145 °C in air on the tensile and flexural mechanical properties of 20% glass fiber (GF)-reinforced commercial grade polybutylene terephthalate (PBT) composites were studied. The results showed that as the aging temperature increased, the tensile and flexural strength of the GF/PBT composites significantly decreased. However, the elastic modulus of the composites was almost independent of aging. As the aging temperature increased, the separation between GF and the PBT matrix became more pronounced, and the fibers exposed on the surface of the matrix became clearer and smoother, indicating a decrease in interfacial adhesion between the PBT matrix and GF. The reason for this decrease in strength and brittle fracture of composites is the interface damage between the GF and PBT matrix caused by the difference in their thermal expansion coefficients during thermal aging.

## 1. Introduction

Polybutylene terephthalate (PBT) is one of the toughest crystalline thermoplastic engineering plastics. It has very good chemical stability, mechanical strength, and electrical insulation [1]. PBT modified with glass fiber (GF) can be used to manufacture electronic parts that require operation in long-term high-temperature conditions with high dimensional stability [2,3,4] and materials with high flame retardancy [5,6]. The change in electrical properties of PBT caused by moisture absorption is very small, the insulation voltage is very high, and the molding stability and dimensional accuracy are excellent; therefore, the material is suitable for production of high-voltage parts [7,8,9]. Due to its good fluidity in the molten state, it is suitable for injection processing of electrical parts with complex structures, such as integrated circuit sockets, printed circuit boards, computer keyboards, electrical switches, fuses, temperature switches, and protectors [10,11].

Outdoor use often encounters various environmental changes, such as damp heat and ultraviolet radiation, requiring anti-damp heat and anti-ultraviolet radiation for the use of these materials. A major disadvantage of PBT is that its instability in hydrolysis and high temperature leads to structural changes in macromolecular chains (especially chain breaks), resulting in a loss of toughness. Therefore, there is only a very narrow processing window (usually 250–260 °C) [12]. Some properties of polymer materials degrade, such as yellowing, loss of gloss, or a significant decline in their mechanical properties when exposed to water, UV radiation, or a harsh environment during processing, storage, and use [13]. These phenomena are referred to as the aging of polymer materials, which leads to limitations in many application fields. In environmental elements, heat and oxygen can usually act on materials at the same time, especially for polymers with relatively low heat resistance and easy oxidation and degradation.

In order to clarify and overcome these shortcomings, it is very important to study the influence of thermal oxidation aging on the properties of polymer composites. Chen et al. [14] conducted accelerated aging experiments on PBT at high temperatures, indicating that during the thermal aging process, phenomena such as terminal hydroxyl oxidation, polymer chain breakage, and the formation of oxidative cross-linking between macromolecules are prone to occur, severely damaging the performance of PBT materials. Zhang et al. [15] studied a series of crystalline PBT materials and analyzed the correlation between their electrical properties and crystallinity. The results showed that as the crystallinity of PBT increased, the surface flashover voltage of the PBT materials increased. Souilem et al. [16] studied the electrical aging performance of PBT using a thermal-stimulated depolarization current method. In another study, the effects of physical aging processes on the electrical and thermal properties of semi-crystalline PBT were investigated [17].

In order to meet the application needs of PBT in industrial equipment, it is necessary to modify the PBT. Controlling the selective distribution of functional fillers in a continuous polymer blend matrix is an effective method for obtaining high-performance functional composites with relatively low filler loading. Wen et al. [18] systematically studied PBT/PC/GNPs composite materials, especially the segregation distribution of GNP functional fillers and the effect of the PBT/PC ratio on the thermal conductivity, conductivity, and mechanical properties of the composite materials. Chiu [19] and Hoeks [20] obtained PBT/PC mixtures, indicating excellent chemical compatibility between the two and effectively increasing the modulus and tensile strength to a certain extent. Bardash et al. [21] established a correlation between the conductivity and processing methods of PBT/MWCNT nanocomposites using multi-walled carbon nanotubes (MWCNTs) as filling materials.

Research on the aging of PBT-based composite materials, hydrolysis, and thermal instability are essential. Jie [22] studied the effects of 120 °C thermal oxidation aging on the static and dynamic mechanical properties, thermal behavior, and morphology of DOPO-PBT composites. The results showed that the mechanical properties declined in the initial stage of aging, while the embrittlement occurred in the late stage. The crosslinking and degradation of PBT molecular chains were the main factors in the entire process of thermal oxidation aging. Hashima [23] studied the tensile stress–strain curve of PBT-LLDPE-SEBS copolymers and the mechanical properties when annealed at 150 °C for 96 h. In addition, Sanchez [24] aged a PC/PBT mixture using natural and accelerated methods and evaluated the tensile and impact properties before and after aging and after recycling. Borukaev [25] used a fractal analysis method to analyze the induction period of PBT + z composites during thermal aging.

PBT-based composites are increasingly being used in structural applications where temperature rise is a common environmental condition; therefore, the thermal aging behavior of composites is of particular interest. In some cases, the temperature may drop suddenly soon after a sudden rise. Fiber-reinforced PBT–matrix composites, especially matrix networks, undergo significant chemical and structural changes during thermal aging. The most common damages that may occur in PBT composites exposed to high temperatures are delamination and microcracks. Hence, the influence of thermal aging on the mechanical properties of GF/PBT composites should be studied in detail. However, in the literature, there are only a few studies that discuss the effects of high temperature on the properties of GF/PBT composites. The aim of this paper is to study the effects of thermal aging at different temperatures on the morphology, mechanical properties, and interface between GFs and a PBT matrix.

## 2. Materials and Methods

### 2.1. Materials and Sample Preparation

**Figure 1:**
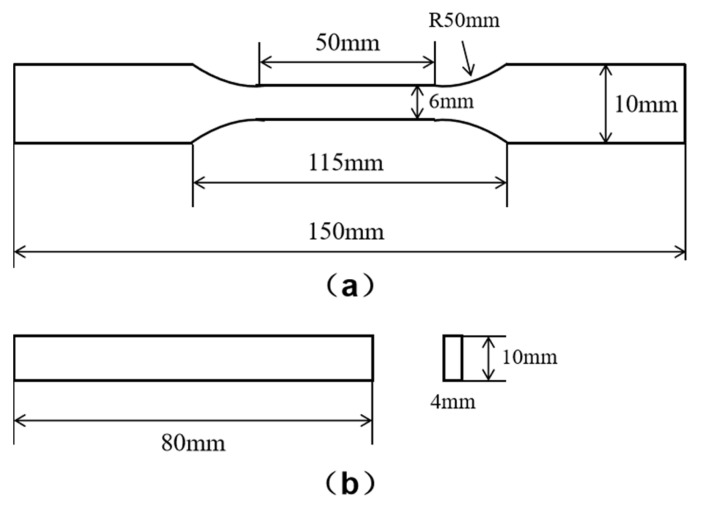
Dimensions of the samples for tensile (**a**) and flexural tests (**b**).

The composite material prepared in this study was a mixture of a PBT matrix with 20% GFs as the reinforcement. The PBT powder was purchased from Honghe Co., Ltd. (Zigong, China), and the FR5301B-2000 GF with an average diameter of 10 μm was purchased from Chongqing Polymer Composite Int. (Chongqing, China). To prepare the fiber-reinforced composites, first, the PBT matrix material was melted and heated to 240 °C. Then, the GF was added into the melted PBT and mixed. The samples for the mechanical tests were made of GF/PBT composite in the form of plates and dog-bone-shaped samples. The dimensions of the dog-bone-shaped samples used for the tensile tests are shown in Figure 1a. In the flexural test, rectangular plate samples were used, as shown in Figure 1b. The support span of the plate samples was 64 mm.

### 2.2. Thermal Aging Procedure

High-temperature aging of the GF/PBT composite samples was carried out using an HZ-2004 (Dongguan Lixian Instrument Scientific Co., Ltd., Dongguan China) furnace under air conditions at various temperatures of 85, 100, 115, 130, and 145 °C for 180 h. These aging parameters were selected to observe the onset of changes in the GF/PBT morphology in the middle of the temperature range, which is between 115 and 130 °C. The samples were placed in the furnace after reaching the required temperature. A reference sample without heat treatment was also prepared for comparison. Here, we design samples as GF/PBT-x, where x indicates the aging temperature. The sample designed as GF/PBT-0 is the reference sample.

### 2.3. Mechanical Testing

#### 2.3.1. Mechanical Properties

Tensile and flexural tests of GF/PBT composites were carried out after heat treatment to study high-temperature damage effects. The dog-bone-shaped samples were used to study the tensile properties. Mechanical testing equipment MTS E45 (MTS, Eden Prairie, MN, USA) was used to carried out the tensile test. For the measurements, the ASTM D3039 [ASTM D3039/3039M-08: Standard Test Method for Tensile Properties of Polymer Matrix Composite Materials, ASTM International, (2008).] standard was used. The test was performed at a constant cross-head speed of 50 mm/min at room temperature. To measure the gauge length region displacement, a 50 mm length extensometer was used. Five samples were tested to calculate the average values. In the test, the elastic modulus, tensile strength, and tensile strain were evaluated.

Rectangular plate samples were used for the flexural test. The flexural performance was studied in three-point bending mode using the ASTM D7264 [Standard test method for flexural properties of polymer matrix composite materials (2015), https://www.astm.org/d7264_d7264m-07.html, (accessed on 16 July 2023)] standard. The MTS E45 testing equipment (MTS, Eden Prairie, MN, USA) was used for flexural tests with a cross-head speed of 1 mm/min. Five samples were tested to calculate the average values.

The elastic modulus E was calculated as the slope tangent to the initial linear portion of the tensile stress–strain curve. The tensile and flexural strength σ_ten_ and σ_fl_, respectively, and the failure strain ε of the composites were calculated using the following formulas:

$$\sigma_{ten}=P/bh\;\;\sigma_{fl}=3PL/2{bh}^{2}\;\;\varepsilon =6Dh/L^{2}$$

where L, b, and h are the span length, width, and thickness of the sample plate, respectively, and P and D are the maximum load and deflection before the failure, respectively.

#### 2.3.2. Surface Morphology

The morphology of the fractured sample surface was analyzed using a scanning electron microscope (SEM) TESCAN MIRA III (Brno, Czech Republic) operated at 5 kV. The samples with fractured surfaces were prepared after the fracture in tensile measurements. Before the SEM study, the samples were coated with carbon film to increase their resistivity using the vapor deposition technique.

## 3. Results

### 3.1. Tensile Properties

**Figure 2:**
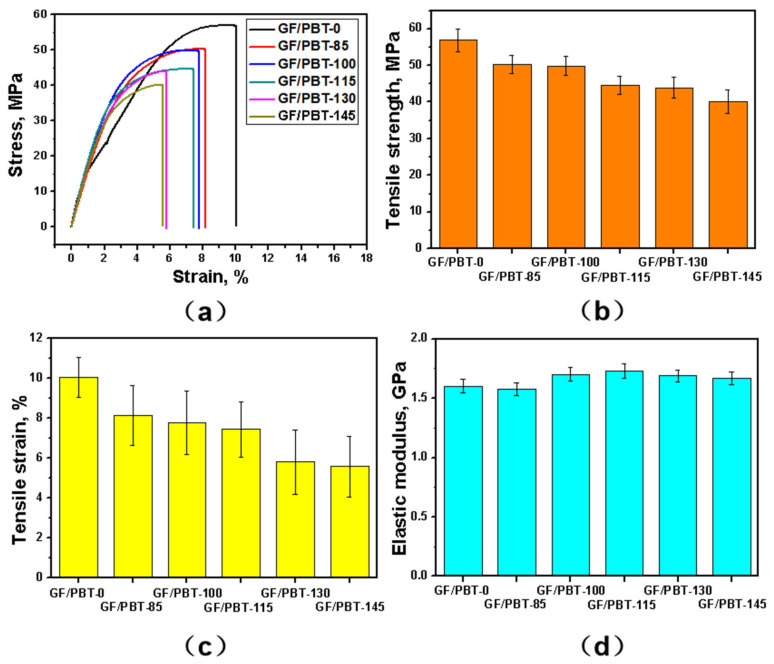
Tensile stress–strain curves of the aged GF/PBT composites (**a**), tensile strength (**b**), tensile strain (**c**), and elastic modulus (**d**) as functions of temperature.

In Figure 2, the tensile characteristics of the GF/PBT composite after aging tests at different temperatures are shown. The heat treatment effect was clearly observed. In Figure 2a the stress–strain curves of the GF/PBT composite after aging at different temperatures are shown. The reference sample GF/PBT-0 demonstrated a different stress–strain curve. At small strains, the sample had a small region of elastic deformation. As the strain increased, a large region with plastic deformation was observed. The composites after aging treatment exhibited elastic deformation at a low strain, followed by plastic deformation and then fracture with a decrease in tensile strength. The tensile strength, strain, and elastic modulus are shown in Figure 2b–d, respectively. In Figure 2b, the tensile strength of the composites decreases with increasing aging treatment temperatures. The tensile strength values of GF/PBT-0, GF/PBT-85, GF/PBT-100, GF/PBT-115, GF/PBT-130, and GF/PBT-145 were 51.8, 50.3, 49.9, 47.0, 43.9, and 40.1 MPa, respectively. Similar to the tensile strength, the tensile strain demonstrated the same behaviour, as shown in Figure 2c. The tensile strain values of GF/PBT-0, GF/PBT-85, GF/PBT-100, GF/PBT-115, GF/PBT-130, and GF/PBT-145 were 10.6%, 8.1%, 7.1%, 6.8%, 5.8%, and 5.6%, respectively. The obtained tensile stress and strain values of the reference sample were comparable to the literature data for 80 Mpa and 5% [26] and 73 Mpa [27]. The observed value mismatch can be explained by a difference in GF parameters. The elastic modulus of the GF/PBT was in the range of 1.55–1.75 Gpa, as shown in Figure 2d, which is also in agreement with the literature data of 1.8 Gpa [28]. Compared to tensile strength and strain, the elastic modulus did not show any dependence on the aging temperature. This can be explained by the fact that, by definition, the elastic modulus is the characteristic of the material in the linear region (low strain) of the stress–strain curve, while tensile stress and strain are the characteristics at the failure point (high strain in the non-linear region) of the stress–strain curve. Therefore, thermal aging can only have a strong impact on the mechanical properties of GF/PBT composites at high strain values.

Degradation of the mechanical properties of the GF/PBT composite can be due to scission and cross-linking of linear molecular chains of the PBT matrix, resulting in brittleness of the composites. The change in mechanical properties can also be explained by increasing the crystallinity of the PBT matrix, which depends on the aging parameters. It should be noted that during the aging treatment, the processes of scission of molecular chains, crosslinking, and crystallinity increase occur simultaneously. It is apparent that the mechanical properties of composites are also determined by the interfacial interaction between the PBT matrix and GFs. As the aging temperature increases, the adhesion between the PBT matrix and GFs can decrease.

### 3.2. Failure Analysis

**Figure 3:**
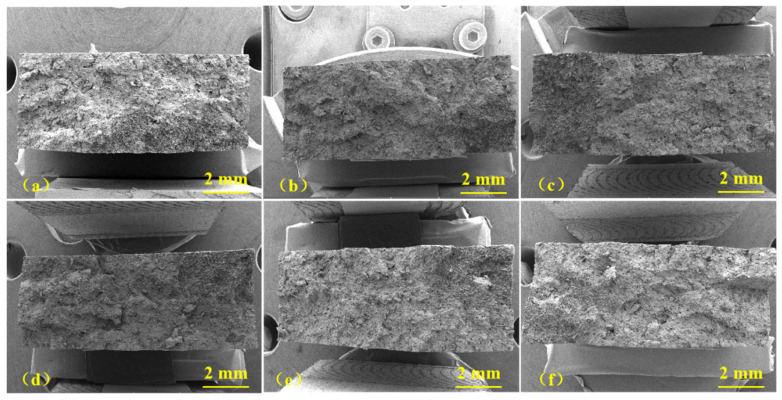
Low-magnification SEM images of the fractured surface of aged samples. (**a**) GF/PBT-0, (**b**) GF/PBT-85, (**c**) GF/PBT-100, (**d**) GF/PBT-115, (**e**) GF/PBT-130, (**f**) GF/PBT-145.

**Figure 4:**
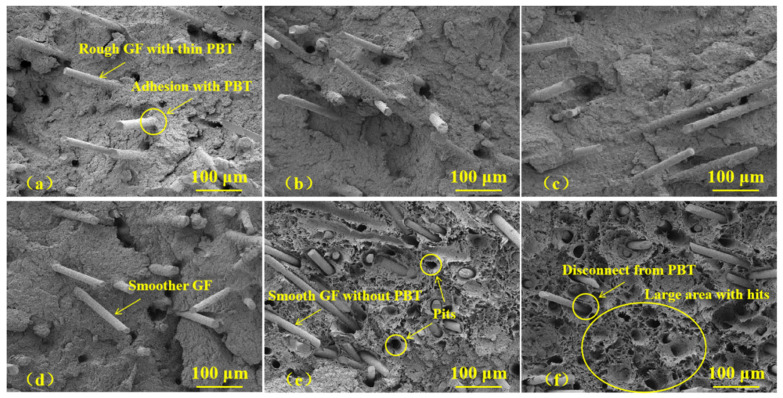
High-magnification SEM images of the fractured surface of aged samples. (**a**) GF/PBT-0, (**b**) GF/PBT-85, (**c**) GF/PBT-100, (**d**) GF/PBT-115, (**e**) GF/PBT-130, (**f**) GF/PBT-145.

The fracture surface before and after heat treatment analyzed using SEM is shown in Figure 3 and Figure 4. Figure 3 shows the overall fracture morphology of the GF/PBT composites. The fracture surface was observed to be rough and uneven. No obvious crack initiation or propagation were observed, indicating that GF/PBT composite samples had high toughness. In addition, in terms of the overall fracture morphology, there was no significant difference between the composite samples aged at different temperatures. Therefore, we captured high-magnification images of the fracture surface, shown in Figure 4, to clarify the failure behavior of the aged composites. The SEM image shows that the GFs were well-embedded in the PBT matrix. In Figure 4a, it can be seen that for the GF/PBT-0 composite sample, a thin PBT coating was found on the surface of the GFs. The adhesion of the fiber matrix was very high, and there was closely adhered PBT at the root of the GF, which also shows that the PBT matrix and the GF had good interfacial adhesion before aging. After aging at 85 and 100 °C, as shown in Figure 4b,c, the GFs were still covered by a thin layer of the PBT matrix. At the same time, most of the fibers were rarely separated and pulled out of the matrix, which indicates that the PBT matrix was well-combined with the fibers and could withstand temperature of 100 °C. However, for the GF/PBT composites aged at 115 °C, it was observed that the surface of the GF was relatively smooth, as shown in Figure 4d. Further increases in the aging temperature to 130 and 145 °C resulted in an obvious separation between the fibers and the matrix, as shown in Figure 4e,f, and the fibers exposed on the surface of the matrix were very clear. At the same time, a large number of pits were left on the surface of the matrix, and the pit density increased with increasing aging temperatures. This fracture morphology is a characteristic of the ductile-failure mode of the matrix [29]. The ductile area is considered as the region of the crack initiation [26]. However, in our experiments, both modes were not observed. The ductile-failure mode replaced the brittle-fracture mode with a relatively flat morphology. Therefore, it can be concluded that the heat treatment of the samples at temperatures above 130 °C can alter the cracking mechanism; however, the details are still unclear. The observed separation phenomenon between the fibers and matrix can be attributed to the difference in the coefficient of thermal expansion (CTE) between the GFs and PBT matrix. The CTE of the pure PBT is 14.4 × 10^−5^ K^−1^ [30], and for the GF, α-quartz CTE can be used, which is in the range of 7.1–13.1 × 10^−6^ K^−1^ [31]. Therefore, there is an order-of-magnitude difference in the CTE values. In the process of thermal aging, the difference in the CTE between the two will produce micro-residual stress, which results in interface damage between the fiber and matrix. The interface damage reduces the interface adhesion and results in a decrease in the tensile strength of the aged composites.

### 3.3. Flexural Properties

**Figure 5:**
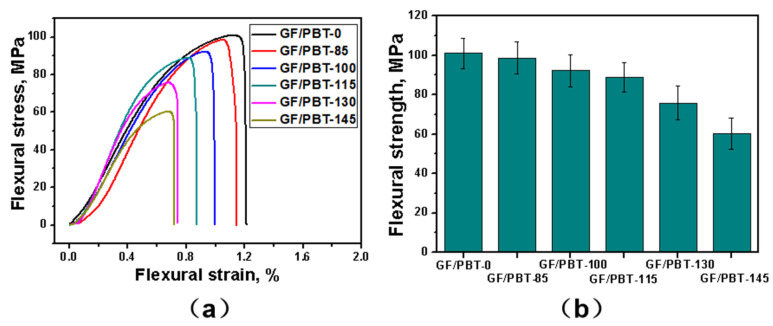
Flexural stress–strain curves (**a**) and flexural strength (**b**) of the aged samples as functions of temperature.

Figure 5a shows the flexural stress–strain curves of the composites before and after aging tests at different temperatures. The reference sample GF/PBT-0 demonstrated the highest flexural strength and strain. The samples after aging showed a decrease in the flexural strength and strain with increasing temperature. Generally, the temperature dependence of the flexural stress–strain curves was similar to the tensile stress–strain curves. In Figure 5b, the flexural strength derived from Figure 5a as a function of the aging temperature is shown. The strength decreased from 100.9 to 60.3 MPa when the aging temperature was increased from 85 to 145 °C. This phenomenon can also be explained by the degradation of the PBT matrix properties (increased brittleness) and the damage of the interface between the GFs and PBT matrix after high temperature treatment, similar to the tensile experiments.

## 4. Conclusions

In summary, GF (20%)-reinforced PBT was aged in the temperature range of 85 to 145 °C to study its mechanical properties. The mechanical properties obtained from the tensile and flexural stress–strain curves and the fracture morphology of the GF/PBT composites after the aging treatment were studied. It was found that as the aging temperature increased, the tensile and flexural strength of the GF/PBT composites significantly decreased, whereas the elastic modulus of the composites did not show any significant change. Scanning electron microscopy revealed that the fractured surface of the samples after aging treatment did not exhibit signs of significant plastic deformations in the temperature range of 85–115 °C, indicating a high brittleness of the aged material. At higher aging temperatures, ductile-failure mode was observed. SEM also revealed damage at the GF/PBT interface, which was caused by a decrease in the adhesion force, leading to a deterioration in the mechanical properties. The damage to the GF/PBT interface at high aging temperatures was explained by the CTE difference between the GFs and PBT.

## Data Availability

Not applicable.
